# Retinal astrocytes transcriptome reveals Cyp1b1 regulates the expression of genes involved in cell adhesion and migration

**DOI:** 10.1371/journal.pone.0231752

**Published:** 2020-04-24

**Authors:** Juliana Falero-Perez, Christine M. Sorenson, Nader Sheibani

**Affiliations:** 1 Department of Ophthalmology and Visual Sciences, University of Wisconsin School of Medicine and Public Health, Madison, Wisconsin, United States of America; 2 McPherson Eye Research Institute, University of Wisconsin School of Medicine and Public Health, Madison, Wisconsin, United States of America; 3 Department of Pediatrics, University of Wisconsin School of Medicine and Public Health, Madison, Wisconsin, United States of America; 4 Department of Biomedical Engineering, University of Wisconsin School of Medicine and Public Health, Madison, Wisconsin, United States of America; 5 Department of Cell and Regenerative Biology, University of Wisconsin School of Medicine and Public Health, Madison, Wisconsin, United States of America; Institute for Advanced bioscience, FRANCE

## Abstract

Astrocytes (AC) are the most abundant cells in the central nervous system. In the retina, astrocytes play important roles in the development and integrity of the retinal neurovasculature. Astrocytes dysfunction contributes to pathogenesis of a variety of neurovascular diseases including diabetic retinopathy. Recent studies have demonstrated the expression of Cyp1b1 in the neurovascular cells of the central nervous system including AC. We recently showed retinal AC constitutively express Cyp1b1, and global Cyp1b1-deficiency (*Cyp1b1*-/-) attenuates retinal ischemia-mediated neovascularization *in vivo* and the pro-angiogenic activity of retinal vascular cells *in vitro*. We also demonstrated that Cyp1b1 expression is a key regulator of retinal AC function. However, the underlying mechanisms involved need further investigation. Here we determined changes in the transcriptome profiles of *Cyp1b1*+/+ and *Cyp1b1*-/- retinal AC by RNA sequencing. We identified 585 differentially expressed genes, whose pathway enrichment analysis revealed the most significant pathways impacted in *Cyp1b1*-/- AC. These genes included those of axon guidance, extracellular matrix proteins and their receptors, cancer, cell adhesion molecules, TGF-β signaling, and the focal adhesion modulation. The expression of a selected set of differentially expressed genes was confirmed by RT-qPCR analysis. To our knowledge, this is the first report of RNAseq investigation of the retinal AC transcriptome and the molecular pathways impacted by Cyp1b1 expression. These results demonstrated an important role for Cyp1b1 expression in the regulation of various retinal AC functions, which are important in neurovascular development and integrity.

## Introduction

The cytochrome P450 superfamily consists of many heme-containing monooxygenases. They are best known for their roles in drug metabolism. CYP1B1 is involved in many processes in the body, such as assisting with reactions that break down drugs and produce certain fats (lipids). It is expressed in both adult and fetal human extrahepatic tissues, including most of the parenchymal and stromal tissues from brain, kidney, prostate, breast, cervix, uterus, ovary, lymph nodes [1], and ocular tissues [2, 3]. Mutations in this enzyme are a risk factor for the development of primary congenital glaucoma in humans [4]. However, the underlying cellular and molecular mechanisms are not fully revealed.

We previously showed expression of Cyp1b1 is essential for ischemia-mediated retinal neovascularization as occurs in retinopathy of prematurity, and the proangiogenic function of retinal vascular cells in culture [5–7]. However, how Cyp1b1 expression impacts these processes remained largely unknown. We showed Cyp1b1 is constitutively expressed in vascular endothelial cells and perivascular supporting cells from vascular beds of many organs including retina [5, 7]. Recently, we also demonstrated that Cyp1b1 is expressed in retinal astrocytes (AC), and *Cyp1b1*-/- retinal AC are more proliferative and migratory [8]. These cells produced increased amounts of fibronectin and its receptors αvβ3 and α5β1 integrins. However, production of inflammatory mediators such as BMP-7 and MCP-1 were decreased in *Cyp1b1*-/- AC. In addition, we observed a significant increase in CD38 expression when *Cyp1b1*-/- AC were challenged with H_2_O_2_ compared with *Cyp1b1*+/+ cells. *Cyp1b1*-/- AC also showed enhanced connexin 43 phosphorylation compared with *Cyp1b1*+/+ cells [8]. Thus, Cyp1b1-deficiency in AC was associated with increased resistance towards oxidative stress.

Astrocytes are the major cell type in the optic nerve head and are vital to the development and maintenance of the retinal astrocytic network and angiogenesis [9, 10]. Under pathological conditions, AC become reactive and contribute to various ocular pathologies including glaucoma and diabetic retinopathy [11]. However, there still much more to delineate regarding Cyp1b1 expression and function in AC. The few studies available to date have confirmed Cyp1b1 expression in AC and neurons, and its upregulation in a variety of gliomas [1, 12]. To our knowledge, we were first to report the impact of Cyp1b1 expression on retinal AC function. However, the intracellular pathways that mediate these activities of Cyp1b1 in AC remain elusive.

RNAseq is one transcriptomic approach where the total complement of RNAs from a given sample is isolated and sequenced using high-throughput technologies [13]. RNAseq technology has the potential to provide very useful, detailed information on the intracellular pathways impacted by Cyp1b1 expression, and identify the networks of genes involved. The purpose of the current study was to utilize this powerful technique to delineate the detailed molecular mechanisms of Cyp1b1 action in retinal AC by determining the changes in patterns of gene expression networks impacted by Cyp1b1 expression. The identification of genes whose expression is affected by the presence or absence of Cyp1b1 will provide additional clues to the intracellular mechanisms of Cyp1b1 action and function in retinal AC. This knowledge should lead to the discovery of new targets for modulation of Cyp1b1 activity and their potential therapeutic use.

## Results

### RNAseq analysis and global gene expression profiles of *Cyp1b1+*/+ and *Cyp1b1-*/- retinal AC

**Fig 1:**
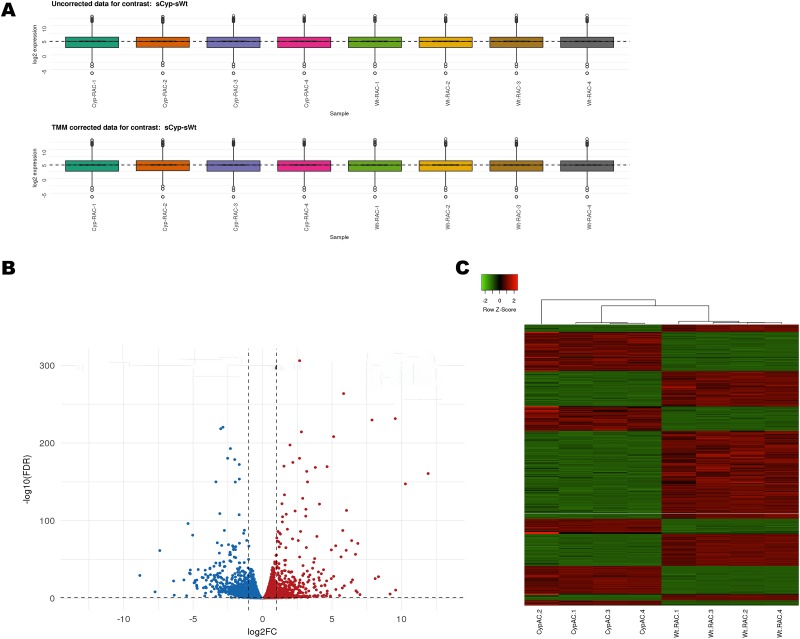
Gene expression profiles of the *Cyp1b1*+/+ and *Cyp1b1*-/- retinal AC. (A) Box plot showing overall TMM expression values for the *Cyp1b1*-/- AC and control samples. (B) Volcano plot showing differentially express genes. For each plot, the X-axis represents log_2_ FC and the Y-axis represents -log_10_ (FDR). The differentially expressed genes (DEG) are shown as red indicating increased expression and blue indicating decreased expression. (C) Hierarchical clustering of DEG. Red indicates increased expression and green indicates decreased expression. The DEG were defined as having absolute FC> 1.5 and an FDR< 0.05.

In order to investigate the impact of Cyp1b1 expression on the transcriptome profile of retinal AC, we performed RNAseq analysis of *Cyp1b1*+/+ and *Cyp1b1*-/- AC. All samples were uniquely barcoded, pooled and sequenced in one lane on an Illumina HiSeq 2500 platform. The average number of reads for *Cyp1b1*+/+ AC was 2.34x10^7^ and for *Cyp1b1*-/- AC was 2.32x10^7^. All sequence reads were mapped to the reference mouse genome using STAR (Spliced Transcripts Alignment to a Reference) [14]. To determine the expression level of various genes, mapped paired-end reads for genes were counted in each sample using RSEM (RNASeq by Expectation Maximization) [15]. Gene expression was normalized by the method of trimmed mean of M-values (TMM), where the product of this factor and the library sizes defines the effective library size [16]. For each sample contrast, simple boxplots per sample expression distributions were constructed before and after TMM normalization (Fig 1A). Overall TMM values were similar in each sample, indicated by the uniform distributions.

Analysis of differentially expressed genes was performed with a glm using the edgeR package [17]. In order to decide which genes are differentially expressed (DEG), the adjusted p-value-not the raw p-value- was defined to be 0.05. To control the false discovery rate (FDR), a Benjamini-Hochberg correction was applied [18]. Fig 1B represents a Volcano plot showing DEG as red and blue dots denoting up- and down-regulated expression, respectively, at an adjusted p-value (FDR) significance threshold of 0.05. The gray dots reflect those genes with no evidence of statistically significant changes in expression. The two solid gray lines denote the boundary of a two-fold change. We also conducted a hierarchical clustering analysis of DEG from all samples with Ward’s method of Euclidean distances [19], and created a heatmap with the heatmap function from Heatmapper: web-enabled heat mapping for all [20]. The results indicated that gene expression was similar in each group (Fig 1C).

**Fig 2:**
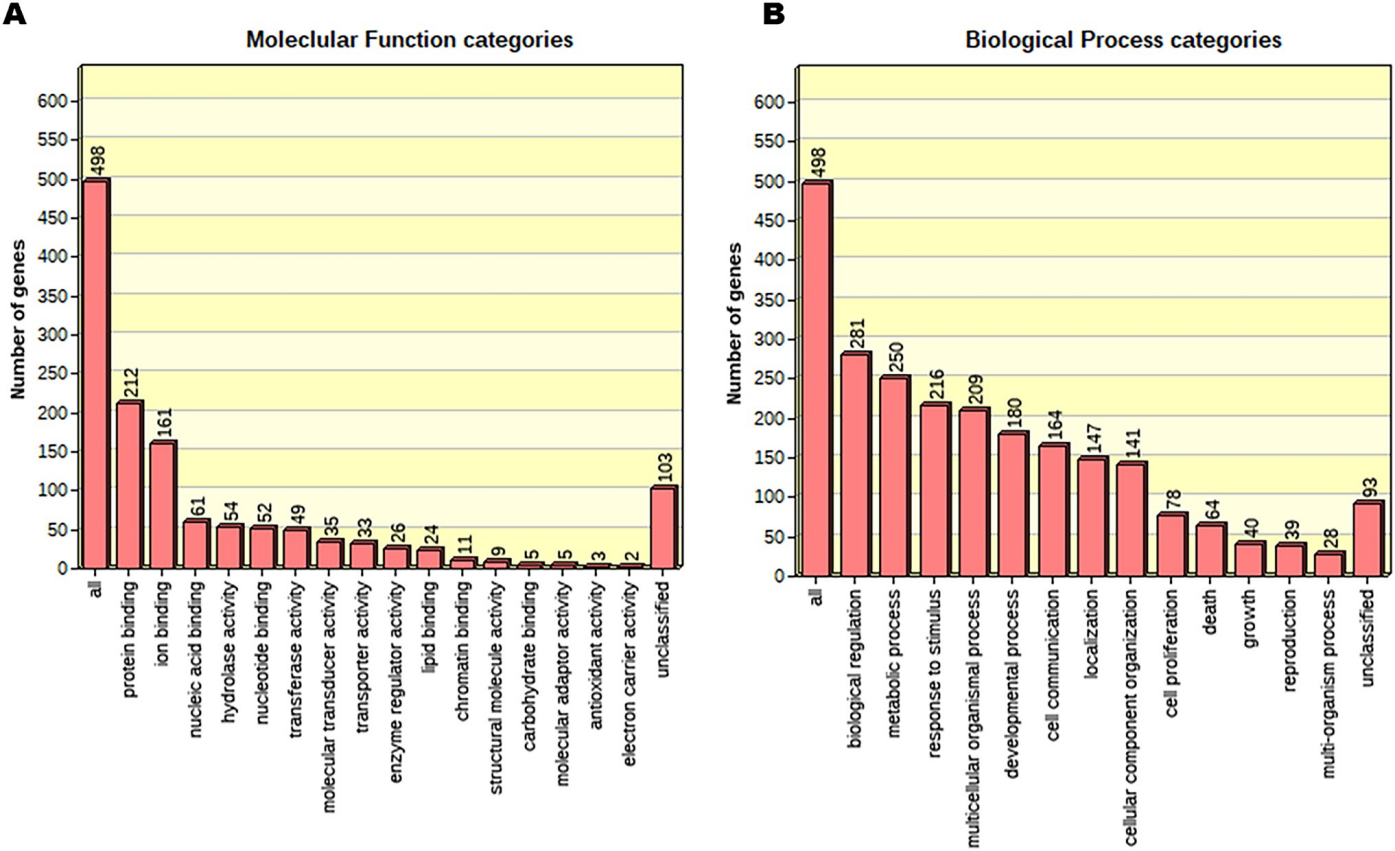
The biological process and molecular functions significantly impacted in *Cyp1b1*-/- AC. (A) Bar graph of the biological process categories. (B) Bar graph of the molecular function. DEG were subjected to GO enrichment analysis having absolute FC> 2 and a FDR< 0.05.

The samples from the *Cyp1b1*+/+ and *Cyp1b1*-/- AC groups were assayed for DEG. The threshold was adjusted to Log2 fold-change with an absolute value of 2.0 and a p-value <1e-7. This yielded 585 transcripts (236 up- and 349-downregulated) for the downstream pathway analysis. The 20 most up- and downregulated genes are listed in Table 1. Within the top upregulated genes, we identified the following genes: Dsp (6.81-fold), Uty (11.92-fold), Cysltr1 (8.34-fold), Cdx2 (6.47-fold) and Kdm5d (10.28-fold). These genes have important roles in different biological and molecular processes including actin-mediated cell contraction, cardiac muscle contraction, oxidoreductase activity, and demethylase activity among others [21–24]. The top downregulated genes including Ptprf (-1.01-fold), Fgf10 (-1.03-fold), Prl2c3 (-1.034), Tgfb3 (-1.04-fold), Mical1 (-1.05-fold), Ndrg2 (-1.05-fold), Ptgs1 (-1.0487-fold), Aplp1 (-1.08-fold), and Steap3 (-1.08-fold) have important roles in processes including cell proliferation, apoptosis, regulation of DNA replication, and metabolic processes among others [25–27]. DEG (585 total) were subjected to Gene Ontology to establish a connection with the biological processes and molecular functions that these genes contribute to (Fig 2).

**Table 1:** Top 20 up- and down-regulated genes in Cyp1b1-/-AC.

Symbol	Description	Log2FC	p-value	FDR
**Up-regulated**				
Eif2s3y	eukaryotic translation initiation factor 2, subunit 3, structural gene Y-linked	12.01499	0	0
Uty	ubiquitously transcribed tetratricopeptide repeat gene, Y chromosome	11.91885	3.6E-164	1.9E-161
Ddx3y	DEAD (Asp-Glu-Ala-Asp) box polypeptide 3, Y-linked	11.79096	0	0
Kdm5d	lysine (K)-specific demethylase 5D	10.28087	8.9E-151	4E-148
Gm43302	predicted gene 43302	9.580986	1.21E-12	2E-11
Rpl15-ps2	ribosomal protein L15, pseudogene 2	9.550535	2.2E-235	3.8E-232
Bmp8b	bone morphogenetic protein 8b	8.847732	0	0
Cysltr1	cysteinyl leukotriene receptor 1	8.342675	1.93E-30	1.16E-28
Gm26760	predicted gene, 26760	8.102196	5.58E-28	2.97E-26
Gm10020	predicted pseudogene 10020	7.874941	1.6E-233	2.5E-230
Gm6969	predicted pseudogene 6969	6.85453	7.82E-74	1.63E-71
Dsp	desmoplakin	6.813457	9.78E-10	1.13E-08
Actg-ps1	actin, gamma, pseudogene 1	6.722348	1.08E-11	1.58E-10
Sgsm1	small G protein signaling modulator 1	6.689262	1.12E-59	1.78E-57
Cdx2	caudal type homeobox 2	6.474141	2.61E-26	1.27E-24
Gm38312	predicted gene, 38312	6.410902	1.62E-77	3.66E-75
Oxct2b	3-oxoacid CoA transferase 2B	6.374568	4.13E-23	1.59E-21
AI593442	expressed sequence AI593442	6.040882	1.6E-116	6E-114
Gm45315	predicted gene 45315	6.018783	7.76E-65	1.3E-62
Adarb2	adenosine deaminase, RNA-specific, B2	5.88212	2.23E-18	6.17E-17
**Down-regulated**				
Ptprf	protein tyrosine phosphatase, receptor type, F	-1.009044	3.978E-53	5.686E-51
Gprc5a	G protein-coupled receptor, family C, group 5, member A	-1.02267	2.718E-12	4.306E-11
Vax2	ventral anterior homeobox 2	-1.025959	5.515E-10	6.507E-09
Fgf10	fibroblast growth factor 10	-1.028563	3.695E-21	1.264E-19
Stard9	START domain containing 9	-1.030828	2.45E-13	4.349E-12
Lmo1	LIM domain only 1	-1.031322	5.887E-17	1.438E-15
Prl2c3	prolactin family 2, subfamily c, member 3	-1.034173	1.076E-22	4.025E-21
Gm27177	predicted gene 27177	-1.034225	3.073E-17	7.67E-16
Tgfb3	transforming growth factor, beta 3	-1.035388	2.308E-12	3.687E-11
Flrt3	fibronectin leucine rich transmembrane protein 3	-1.038403	8.71E-09	8.981E-08
Acsf2	acyl-CoA synthetase family member 2	-1.040813	9.692E-12	1.426E-10
Ppic	peptidylprolyl isomerase C	-1.042228	5.977E-32	3.847E-30
Eif3j1	eukaryotic translation initiation factor 3, subunit J1	-1.042647	4.709E-42	5.158E-40
Mical1	microtubule associated monooxygenase, calponin and LIM domain containing 1	-1.046611	1.687E-09	1.899E-08
Ndrg2	N-myc downstream regulated gene 2	-1.047743	2.791E-13	4.941E-12
Ptgs1	prostaglandin-endoperoxide synthase 1	-1.0487	5.856E-29	3.273E-27
Cbr3	carbonyl reductase 3	-1.050294	2.114E-16	4.933E-15
Slfn8	schlafen 8	-1.061261	4.262E-23	1.64E-21
Aplp1	amyloid beta (A4) precursor-like protein 1	-1.075804	8.932E-14	1.662E-12
Steap3	STEAP family member 3	-1.08044	5.467E-14	1.046E-12

The cutoff criteria for this list was any DEG with a FC> 5.88 for upregulation and FC> -1.009 for downregulation.

Pathway enrichment analysis, conducted using KEGG (Kyoto Encyclopedia of Genes and Genomes) [28–30] as a mapping database, 57 pathways were identified with significance level of 0.05. To cut down on the list the P< 0.002 was applied. The pathways with multiple points of commonality and overlap among the Axon guidance, extracellular matrix-receptor interactions, pathways in cancer, cell adhesion molecules, TGF-β signaling pathways, and the focal adhesion were identified as most significantly enriched pathways (Table 2).

**Table 2:** Pathway enrichment analysis of significantly changed genes.

KEGG Pathway name	Pathway Rank	GENES	FDR adjusted enrichment score p-value
Axon guidance	1	Sema3c, Unc5d, Sema5a, Sema3g, Ngef, Lrrc4c, Sema6b, Cxcl12, Unc5c, Sema4f, Plxnb1, Sema6a	3.32e-07
ECM-receptor interaction	2	Itga1, Col1a2, Lama2, Tnxb, Spp1, Lama5, Col5a3, Npnt, Thbs2	4.79e-06
Pathways in cancer	3	Dapk1, Arnt2, Wnt5b, Lama2, Pgf, Hhip, Apc2, Fgf7, Pax8, Lama5, Wnt4, Tgfb3, Ar, Mmp2, Fgf10	1.31e-05
Cell adhesion molecules (CAMs)	4	Vcan, Cdh4, Cd80, Cldn1, Vcam1, Cd34, Ptprf, Cdh3, Icam1	0.0002
TGF-beta signaling pathway	5	Tgfb3, Gdf5, Fst, Lefty1, Bmp7, Bmp8b, Thbs2	0.0002
Focal adhesion	6	Itga1, Col1a2,Lama2,Pgf, Tnxb, Spp1, Lama5, Col5a3, Myl12b,Thbs2	0.0002
Neuroactive ligand-receptor interaction	7	Lepr, Adra1d, Htr1b, Gria4, Grid2, Htr2a, Cysltr1,Drd4, Calcrl, Grid1	0.0020
Metabolic pathways	8	Mgat3, Itpkb, Cda, Xylb, Pla2g4b, Abat, Ckb, Ass1, Isyna1, Aldh1a7, Aldh1a1, Nnmt, Cbr2, B3gnt3, Cbr3, Gldc, Eno2, Ptgs1, Bdh2, Ldhb, Gatm, St3gal1, Sardh	0.0041
Calcium signaling pathway	9	Adcy7, Cacna1d, Itpkb, Cacna1h, Adra1d, Htr2a, Cysltr1	0.0058
Cytokine-cytokine receptor interaction	10	Lepr, Ccl7, Bmp7, Tgfb3, Tnfsf10, Cxcl11, Cxcl12, Gdf5	0.0077
MAPK signaling pathway	11	Mapk13, Tgfb3, Cacna1d, Cacna1h, Pla2g4b, Fgf10, Fgf7	0.0277

**Significantly changed transcriptome**. Column 1 lists the canonical KEGG pathway name, column 2 lists the pathway enrichment score rank in terms of p-value determined by hypergeometric test, column 3 lists the genes that mapped to the KEGG pathway, and column 4 shows the FDR adjusted p-value of significance of the pathway enrichment score.

**Fig 3:**
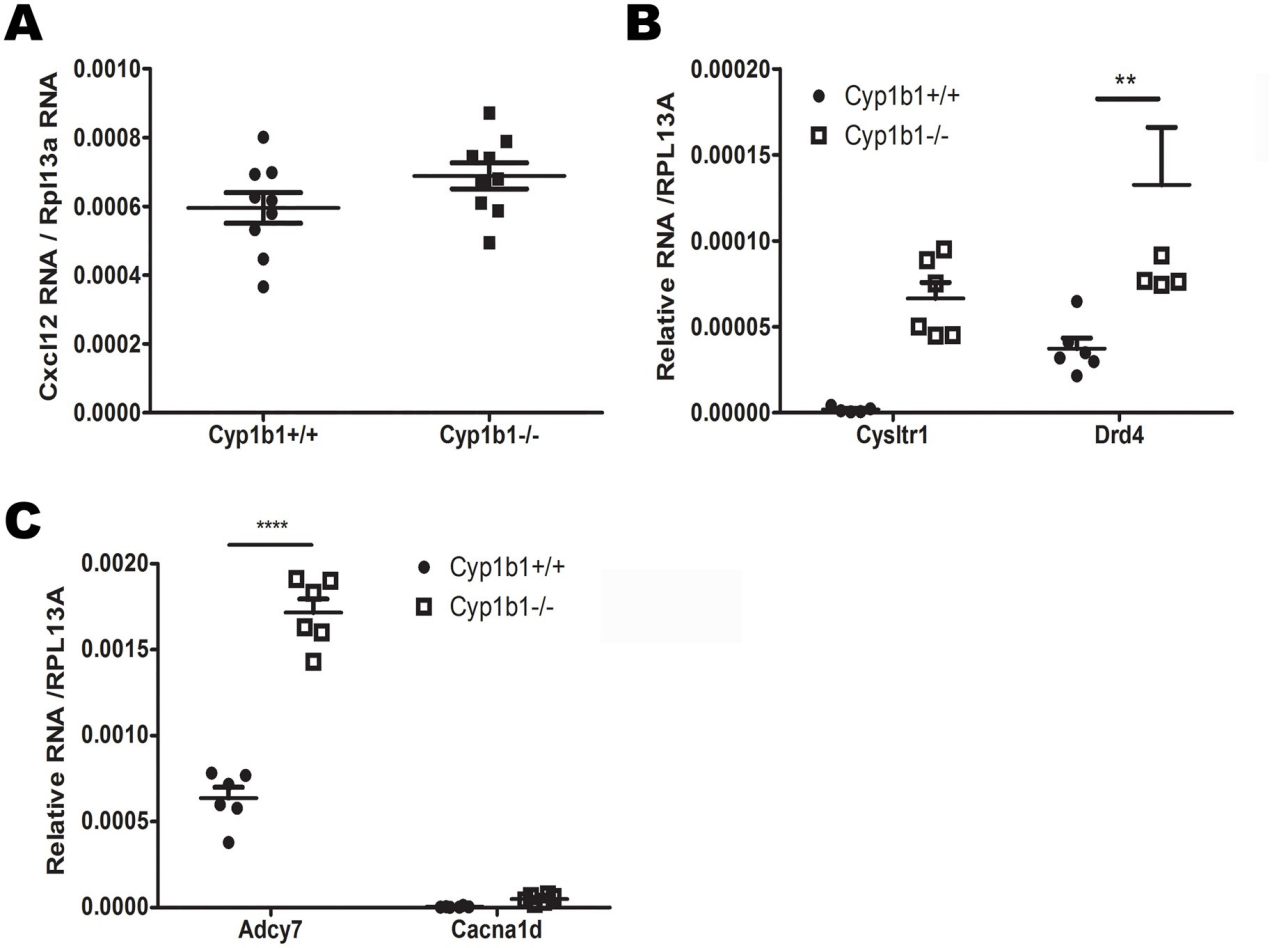
RT-qPCR validation of DEG from different pathways. (A) Genes related to the axon guidance pathway. (B) Genes related to the Neuroactive ligand-receptor interaction pathway. (C) Genes related to the Calcium signaling pathway. (**P<0.01, ****P<0.0001, n≥ 6).

**Fig 4:**
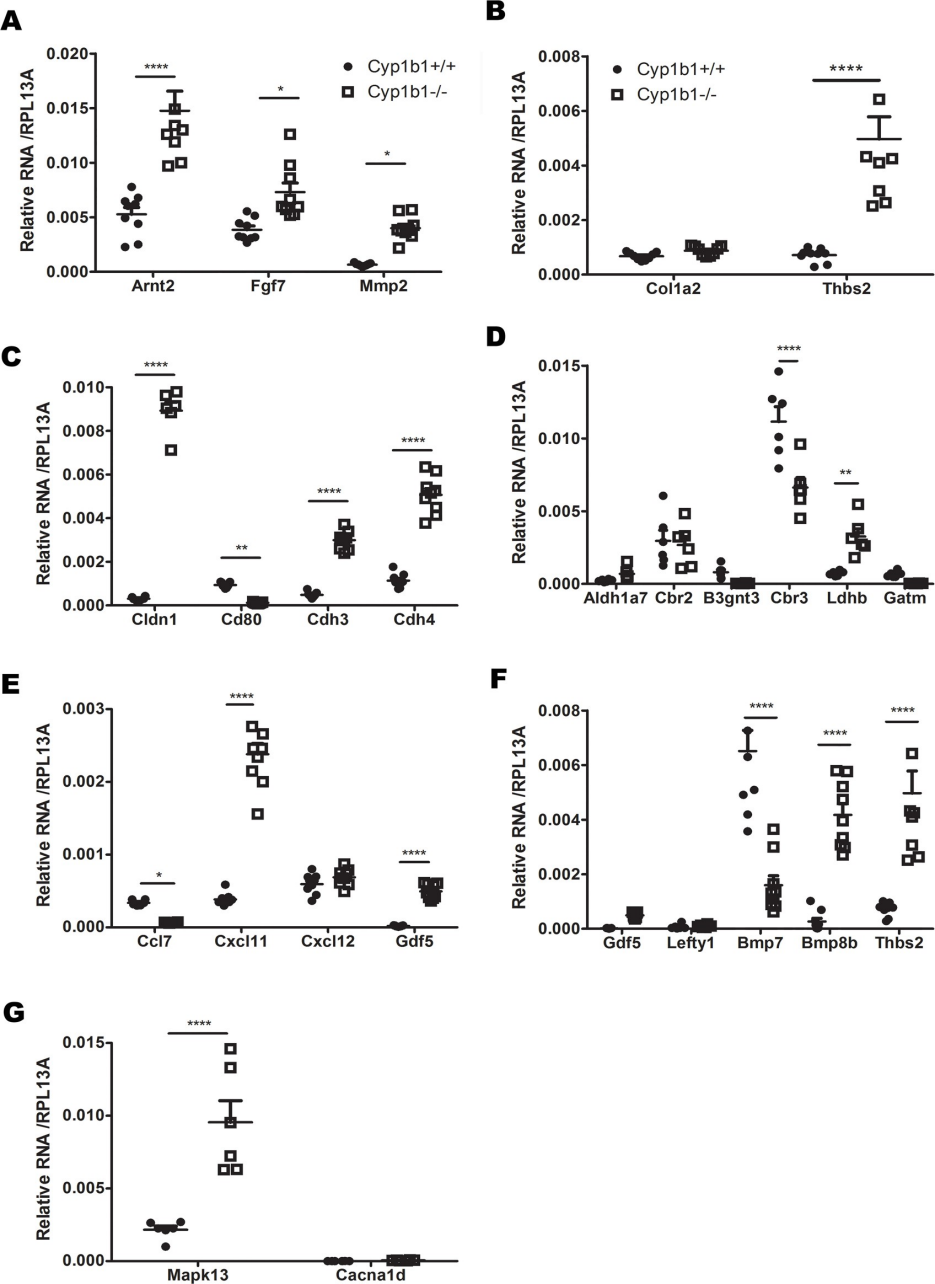
RT-qPCR validation of DEG from different pathways. (A) Genes related to the Cancer pathway. (B) Genes related to the ECM-Receptor and Focal adhesion pathways. (C) Genes related to the Cell adhesion molecules pathways. (D) Genes related to the metabolic pathways. (E) Genes related to the Cytokine-Cytokine interaction pathways. (F) Genes related to the TGF-β signaling pathway. (G) Genes related to the MAPK signaling pathways. (*P<0.05, **P<0.01, ****P<0.0001, n≥ 6).

To validate the RNAseq findings, RT-qPCR were performed on newly extracted RNA from *Cyp1b1*+/+AC and *Cyp1b1*-/-AC. From each KEGG pathway, we selected key genes with important roles in angiogenesis, apoptosis, cell proliferation, cell migration, metabolism and inflammation. We selected 27 genes (Cxcl12, Cxcl11, Col1a2, Arnt2, Fgf7, Mmp2, Bmp7, Thsb2, Bmp8b, Cdh3, Cdh4, Gdf5, Cd80, Cldn1, Lefty1, Cysltr1, Drd4, Aldh1a7, Cbr2, B3gnt3, Cbr3, Ldhb, Gatm, Adcy7, Cacna1d, Ccl7, and Mapk13) involved in the pathways identified in Table 2. We examined changes in their expression by RT-qPCR (Figs 3 and 4). The results obtained with RT-qPCR showed similar changes in expression as those obtained from the RNAseq studies (Table 3). However, the changes in the expression of a number of these genes did not reach significant levels. These included Cxcl12, Cysltr1, Cacna1d, Col1a2, Aldh1a7, Cbr2, B3gnt3, Gatm, and Lefty1.

**Table 3:** Differentially expressed gene selected for RT-qPCR validation.

KEGG pathway name	Symbol	Gene Description	Log_2_FC	p-value
**Axon Guidance**	Cxcl12	chemokine (C-X-C motif) ligand 12	1.10	2.39E-89
**ECM-receptor interaction**	Col1a2	collagen, type I, alpha 2	5.74	1.69E-15
	Thbs2	thrombospondin 2	2.67	4.62E-12
**Pathways in cancer**	Arnt2	aryl hydrocarbon receptor nuclear translocator 2	1.70	1.28E-111
	Fgf7	fibroblast growth factor 7	1.28	6.02E-86
	Mmp2	matrix metallopeptidase 2	5.28	1.77E-13
**Cell adhesion molecules**	Cdh4	cadherin 4 (retinal)	3.23	2.45E-153
	Cd80	CD80 antigen	-3.52	7.99E-27
	Cldn1	claudin 1	4.64	4.05E-173
	Cdh3	cadherin 3 (placental)	3.75	3.80E-54
**TGF-β signaling**	Gdf5	growth differentiation factor 5	4.65	2.08E-11
	Lefty1	left right determination factor 1	2.62	1.32E-12
	Bmp7	bone morphogenetic protein 7	-2.31	2.04E-59
	Bmp8b	bone morphogenetic protein 8b	8.85	0
	Thbs2	thrombospondin 2	2.67	4.62E-12
**Focal adhesion**	Col1a2	collagen, type I, alpha 2	5.74	1.69E-15
	Thbs2	thrombospondin 2	2.67	4.62E-12
**Neuroactive ligand- receptor interaction**	Cysltr1	cysteinyl leukotriene receptor 1	8.34	1.93E-30
	Drd4	dopamine receptor D4	3.32	1.86E-11
**Metabolic pathways**	Aldh1a7	aldehyde dehydrogenase family 1, subfamily A7	3.34	1.25E-25
	Cbr2	carbonyl reductase 2	2.20	3.12E-10
	B3gnt3	UDP-GlcNAc:betaGal beta-1,3-N-acetylglucosaminyltransferase 3	-5.21	
	Cbr3	carbonyl reductase 3	-1.05	2.114E-16
	Ldhb	lactate dehydrogenase B	2.15	7.57E-21
	Gatm	glycine amidinotransferase (L-arginine:glycine amidinotransferase)	-4.63	6.11E-40
**Calcium signaling**	Adcy7	adenylate cyclase 7	1.34	2.44E-15
	Cacna1d	calcium channel, voltage-dependent, L type, alpha 1D subunit	3.87	5.65E-13
	Cysltr1	cysteinyl leukotriene receptor 1	8.34	1.93E-30
**Cytokine-cytokine receptor interaction**	Ccl7	chemokine (C-C motif) ligand 7	-2.40	9.08E-10
	Bmp7	bone morphogenetic protein 7	-2.31	2.04E-59
	Cxcl11	chemokine (C-X-C motif) ligand 11	2.96	2.05E-20
	Cxcl12	chemokine (C-X-C motif) ligand 12	1.10	2.39E-89
	Gdf5	growth differentiation factor 5	4.65	2.08E-11
**MAPK signaling**	Mapk13 (P38-delta)	mitogen-activated protein kinase 13	1.66	5.24E-52
	Cacna1d	calcium channel, voltage-dependent, L type, alpha 1D subunit	3.87	5.65E-13
	Fgf7	fibroblast growth factor 7	1.28	6.02E-86

The expression of selected genes related to the KEGG pathways were validated by RT-qPCR. LogFC and p-values were obtained from RNAseq analysis.

## Discussion

In this study we used RNAseq analysis to determine the global transcriptome profile of *Cyp1b1*+/+ and *Cyp1b1*-/- AC from mouse retina. We identified 585 DEG, whose pathway analysis revealed the most significant biological functions. These included the Axon guidance, extracellular matrix (ECM)-receptor interactions, pathways in cancer, cell adhesion molecules, TGF-β signaling, and the focal adhesion regulation. We also found that some of the top downregulated genes were involved in biological and molecular processes including actin-mediated cell contraction, cardiac muscle contraction, cell proliferation, apoptosis and metabolic processes.

Our findings here were consistent with the results of our previous studies showing increased proliferation and migration of *Cyp1b1-/-* AC. Activation of AC proliferation and migration is important in repair of injuries in the central nervous system (CNS) and scar formation [31, 32]. AC migration is regulated by various factors, among which transforming growth factor-β (TGF-β) plays an important role [33]. In AC, TGF-β suppresses cell proliferation by inducing p15^INK4B^ expression in a Smad3-dependent manner [34]. This is consistent with our findings that showed the downregulation of TGF-β in *Cyp1b1*-/- AC. The upregulation of Smad genes in *Cyp1b1* -/- AC was associated with the enhanced pathways in axon guidance and cell proliferation.

Our results showed changes in Cxcl12, (also known as SDF-1). Cxcl12 is one of the most studied chemokines that induce cell proliferation and migration by binding to its receptor. Under normal conditions, Cxcl12 expression in the CNS is relatively low. However, its expression is upregulated when the CNS is affected by trauma, ischemia, inflammation or infection [35]. Enhanced Cxcl12 expression promotes proliferation of radial glia like cells after traumatic brain injury in rats [36]. Others have shown its therapeutic value by promoting autophagy and migration via PI3K-AKT-mTOR pathway [37].

We recently showed that Cyp1b1 deficiency affects retinal AC ECM production and expression of integrin receptors [8]. We also showed upregulation of cadherins, laminin, and tenascin. These molecules are well known for their roles in cell adhesion [38], and were associated with changes in adhesion observed in *Cyp1b1*-/- retinal AC [8]. Cadherin-4 (also known as R-cadherin) is involved in retinal angiogenesis during development. Dorrell et al. [39] used antibodies or peptides to neutralize R-cadherin, which prevented the normal formation of the retinal vascular network in newborn mice. They also showed that R-cadherin plays a crucial role in the endothelial–astrocyte interactions [39, 40]. Thus, Cyp1b1 expression may impact AC interactions with EC.

Oxidative stress is implicated in many neurodegenerative diseases. Cytochrome P450 activities are generally involved in ROS production due to their involvement in the metabolism of steroids, fat-soluble vitamins, fatty acids, eicosanoids, drugs, carcinogens, and other xenobiotic chemicals [41–43]. CYP enzymes can generate superoxide and hydrogen peroxide through uncoupling reactions, for more details see [44]. We have demonstrated an important role for Cyp1b1 as a modulator of cellular redox state [45]. Studies utilizing vascular cells derived from *Cyp1b1*-/- mice showed an increase in oxidative stress in vascular endothelial cells and perivascular supporting cells [5, 7, 46]. In contrast, *Cyp1b1*-/- retinal AC did not show an increase in oxidative stress compared to *Cyp1b1*+/+ cells under basal conditions [8]. This is consistent with minimal changes in expression of genes that affect cellular redox state in the absence of Cyp1b1. However, incubation of these cells with known inducers of oxidative stress could reveal changes in genes that modulate oxidative stress. This notion is supported by our studies demonstrating that *Cyp1b1*-/- AC elicit a significantly more robust response in expression of CD38 when challenged with H_2_O_2_ compared with *Cyp1b1*+/+ cells [47]. Thus, the elucidation of the mechanisms behind AC resilience to oxidative stress, especially in the absence of Cyp1b1, needs further investigation.

Cytochrome P450 enzymes are involved in metabolism of drugs, and are major source of variability in drug pharmacokinetics and responses. However, in some cases they can also activate compounds consumed in food, converting pro-carcinogens to carcinogens [48]. CYP proteins, involved in steroid or retinoic acid metabolism, could promote or suppress tumors development through hormonal control [49, 50]. Genetic variability could play a role if a polymorphism affected a CYP protein involved in such processes [51]. Our study found multiple genes in the cancer pathway that are associates with the progression or suppression of cancer such as laminin, Tgfβ3, Hhip, Arnt2 and Mmp2 [52–54]. Mmp2 was upregulated in this pathway and is implicated in cancer cell migration [55]. A previous study using a microarray analysis, demonstrated that the increased expression of Mmp2 is involved in invasiveness of malignant glioma [56, 57], an observation that is consistent with our findings. Thus, Cyp1b1 expression has an important role in modulating AC migration by suppressing Mmp2 expression.

A limitation of our studies is the use of cells prepared from mix genders. Our initial in vivo and in vitro vascular cell culture studies did not demonstrate a gender bias in the noted phenotypes with *Cyp1b1*-deficiency. However, our gene expression studies here showed some of the differentially expressed genes in Cyp1b1 null cells are sex linked: Uty; Y-chromosome, Cysltr1; X-chromosome, and Kdm5d; Y-chromosome. Thus, Cyp1b1-deficiency impact on gene expression, and likely noted phenotypes, could be deferentially impacted by gender. Future studies will further address the gender contributions to various phenotypes noted with *Cyp1b1*-deficiecny.

In summary, RNAseq technology was used to investigate the transcriptome profiles of retinal AC and how Cyp1b1 expression modulates their cellular functions. A pathway analysis of DEG indicated the most significantly enriched pathways included Axon guidance, ECM-receptor interactions, as well as cancer and other pathways (cell proliferation, focal adhesion, and cell adhesion). Our transcriptomic approach in this investigation, which relied on RNAseq, was powerful and effective way to allow us to obtain a global view of genes whose expression are impacted by Cyp1b1, likely in a gender dependent manner.

## Materials and methods

### Ethics statement

All animal experiments were performed in accordance to the Association for Research in Vision and Ophthalmology Statement for the Use of Animals in Ophthalmic and Vision Research and were approved by the Institutional Animal Care and Use Committee of the University of Wisconsin School of Medicine and Public Health (the assurance number A3368-01). Animals were sacrificed according to an approved protocol by CO2 asphyxiation.

### Isolation and culture of Cyp1b1-/- retinal AC

Retinal AC were isolated from mouse retina by collecting retinas from one litter of 4-week-old (6 to 7- mix gender) mice using a dissecting microscope, as previously described by us with greater than 98% purity [8, 58]. Briefly, retinas (12 to 14) were rinsed with serum-free Dulbecco’s Modified Eagle’s Medium (DMEM), pooled in a 60 mm dish, minced and digested for 45 min with collagenase Type I (1 mg/ml; Worthington, Lakewood, NJ) in serum-free DMEM at 37°C. Cells were rinsed in DMEM containing 10% fetal bovine serum (FBS) and centrifuged for 5 min at 400 xg. Digested cells were rinsed again in DMEM containing 10% FBS and filtered through a double layer of sterile 40 μm nylon mesh (Sefar America Inc., Fisher Scientific, Hanover Park, IL). Cells were centrifuged for 5 min at 400 xg and medium was aspirated. Cells were washed twice with DMEM containing 10% FBS, resuspended in 1 ml of DMEM containing 10% FBS in a 1.5 ml microfuge tube and incubated with rat-anti-mouse CD31 (Mec13.3; BD Biosciences) coated with sheep anti-rat magnetic beads, and were gently rocked for 1 h at 4°C. Using a Dynal magnetic tube holder, cells not bound to magnetic beads were collected and washed in DMEM containing 10% FBS. Cells were plated in growth medium in a single well of a 24 well plate coated with human fibronectin (2 μg/ml in serum-free DMEM; BD Biosciences, Bedford, MA), and incubated at 33°C with 5% CO2. The cells bound to magnetic beads are generally used to remove retinal EC as we described [58]. Retinal AC were grown in DMEM containing 10% FBS, 2 mM L-glutamine, 2 mM sodium pyruvate, 20 mM HEPES, 1% nonessential amino acids, 100 μg/ml streptomycin, 100 U/ml penicillin, freshly added heparin at 55 U/ml (Sigma, St. Louis, MO), endothelial growth supplement 100 μg/ml (Sigma), and the murine recombinant interferon-γ (R&D, Minneapolis, MN) at 44 U/ml. Cells were maintained at 33°C with 5% CO2. Cells were progressively passed to larger plates, maintained, and propagated in 1% gelatin-coated 60 mm dishes. For all experiments, cells were used at 70–80% confluence unless stated otherwise.

### RNA purification

Total RNA from Cyp1b1+/+ and Cyp1b1-/- retinal AC was purified using RNeasy Mini kit according to manufacturer’s protocol with the DNAse treatment step to eliminate traces of genomic DNA (Qiagen, Germantown, MD). The quality and quantity of the total RNA were measured using an Agilent Model 2100 Bioanalyzer, and samples showing a RIN >8 were selected for further analysis. Samples were stored in RNase-free water and kept at -80°C until further processing.

### RNA sequencing

Eight samples of purified RNA (4 from *Cyp1b1*+/+ retinal AC and 4 from *Cyp1b1* -/- retinal AC) were subsequently subjected to a double round of poly-A mRNA purification, fragmented, and primed for cDNA library synthesis using the TruSeq RNA sample preparation kit (RS-122-9004). All procedures were carried out according to the manufacturer’s instructions (Illumina, San Diego, CA). Following validation (Agilent 2100 Bioanalyzer, DNA 1000) and normalization, samples were clustered (TruSeq pairedend cluster kit v3-cBot-HS, PE-401-3001) followed by paired-end sequencing (100 bp; TruSeq SBS kit v3-HS 200 cycles, FC-401-3001) on a HiSeq2500. The following quality control statistics were used to evaluate the technical quality of the experiments; (1) combined per cycle base quality, (2) per cycle base frequencies, (3) per cycle average base quality, (4) relative 3k-mer diversity, (5) Phred quality distribution, (6) mean quality distribution, (7) read length distribution and (8) read occurrence distribution using the trimming software skewer [59]. Low-abundance genes with a read count below a threshold of 1.0 in two or more samples were excluded. To compare gene expression between Cyp1b1+/+AC and Cyp1b1-/-AC, samples were normalized by trimmed mean of M-values (TMM) using Edge R (version 2.5 of Bioconductor) software [16]. After the inspection of preliminary data, transcript reads were aligned to the preassembled selected reference genome sequence using STAR (Spliced Transcripts Alignment to a Reference) using the default settings [14]. Transcript abundance were performed by using RSEM (RNASeq by Expectation Maximization) [15]. Subsequently, differential analysis of significant changes in gene expression was performed with a glm using the edgeR package [17] in the different genotype pairs (e.g. *Cyp1b1*+/+ vs. *Cyp1b1* -/-). All sequence data have been deposited in the Gene Expression Omnibus with accession number GSE145103.

### Quantitative RT-PCR

Total RNA from retinal AC was extracted using mirVana PARIS kit (Invitrogen). The cDNA synthesis was performed from 1 μg of total RNA using Sprint RT Complete-Double PrePrimed kit (Clontech, Mountain View, CA). One μl of each cDNA (dilution 1∶10) was used as template in qPCR assays, performed in triplicate of three biological replicates on Mastercycler Realplex (Eppendorf) using the SYBR-Green qPCR Premix (Clontech). Amplification parameters were as follows: 95°C for 2 min; 40 cycles of amplification (95°C for 15 sec, 60°C for 40 sec); dissociation curve step (95°C for 15 sec, 60°C for 15 sec, 95°C for 15 sec). Standard curves were generated from known quantities for each of the target gene of linearized plasmid DNA. Ten times dilution series were used for each known target, which were amplified using SYBR-Green qPCR. The linear regression line for ng of DNA was determined from relative fluorescent units (RFU) at a threshold fluorescence value (Ct) to quantify gene targets from cell extracts by comparing the RFU at the Ct to the standard curve, normalized by the simultaneous amplification of RpL13a, a housekeeping gene. All primer sequence used are listed in Table 4.

**Table 4:** Primers to validate differentially express genes.

Gene	Amplicon size (bp)	primer	Primer Sequence (5'->3')	Length	Gene accession
**Cxcl12**	116	F	ctgtgcccttcagattgttg	20	NM_001012477.2
		R	ctctgcgccccttgttta	18	
**Cxcl11**	93	F	tgctgagatgaacaggaaggt	21	NM_019494.1
		R	cgcccctgtttgaacataag	20	
**Col1a2**	69	F	ctggtgcacagggtgtga	18	NM_007743.3
		R	ctcctgcttgacctggagtt	20	
**Arnt2**	104	F	tgcacttcggaaaactccat	20	NM_007488.3
		R	cgagagcccatacacatgc	19	
**Fgf7**	78	F	ttactccatagttctgcaaccagt	24	NM_008008.4
		R	tgttgcccttcccttcataa	20	
**Mmp2**	75	F	gggcttctgtcctgacca	18	NM_008610.3
		R	aagttcttggtgtaggtgtagatcg	25	
**Bmp8b**	70	F	ctgtatgaactccaccaaccac	22	NM_028189.3
		R	ggggatgatatctggcttca	20	
**Cdh3**	75	F	aggcccagctaacacatgac	20	NM_001037809.5
		R	acaaggccacggtgtctc	18	
**Cdh4**	60	F	ttcctggctgctgacaatg	19	NM_009867.3
		R	gtagatctgcagggtcccagt	21	
**Gdf5**	73	F	tttattgacaaagggcaagatg	22	NM_008109.3
		R	aggcactgatgtcaaacacg	20	
**Cd80**	127	F	ttcgtctttcacaagtgtcttca	23	NM_009855.2
		R	tgccagtagattcggtcttca	21	
**Cldn1**	92	F	actccttgctgaatctgaacagt	23	NM_016674.4
		R	ggacacaaagattgcgatcag	21	
**Lefty1**	92	F	actcagtatgtggccctgcta	21	NM_010094.4
		R	aacctgcctgccacctct	18	
**Cysltr1**	95	F	aaggtgctgaggtaccagatagag	24	NM_021476.5
		R	aatcacagcccttgagaagc	20	
**Drd4**	137	F	cccaccaactacttcatcgtg	21	NM_007878
		R	gccatgagcgtgtcacag	18	
**Aldh1a7**	85	F	gtttgcagatgccgacttg	19	NM_011921.2
		R	cgctgcaacacaaatctgac	20	
**Cbr2**	109	F	gcccatgtcacctttcctaa	20	NM_007621.2
		R	ttacccggatcttgtgtgg	19	
**B3gnt3**	115	F	gcaaatacaaccgactgctg	20	NM_028189.3
		R	cactccaggaaaaggacctg	20	
**Cbr3**	60	F	aacgttagcgggagagatga	20	NM_173047.3
		R	cccttgatgtgggaaagaatc	21	
**Ldhb**	86	F	gtagtgggcgttggacaagt	20	NM_001316322.1
		R	acatccaccagggcaagtt	19	
**Gatm**	103	F	ggtgcactacatcggctctc	20	NM_025961.5
		R	acaggaatttcgggaggaa	19	
**Cacna1d**	60	F	gaagctgcttgaccaagttgt	21	NM_001302637.1
		R	aacttccccacggttacctc	20	
**Ccl7**	91	F	ttctgtgcctgctgctcata	20	NM_013654.3
		R	ttgacatagcagcatgtggat	21	
**MAPK13**	63	F	caggctggccttgagtctt	19	NM_011950.2
		R	ccagggctacacagtaagatcc	22	
**Adcy7**		F	gagccttccagacgtccat	19	NM_007406.2
	70	R	aggaggataacggcattgg	19	

### Biological interaction network and KEGG pathway enrichment analysis

Pathway Enrichment Analysis was performed with WEBGESTALT web analysis software (http://bioinfo.vanderbilt.edu/wg2/) by mapping significantly changed genes to corresponding KEGG enrichment pathways, Gene Ontology enrichment and conducting a hypergeometric statistical test with significant level <0.05 after multiple testing correction [60–62]. Significance for pathway level enrichment was defined as having an enrichment score False Discovery Rate (FDR) corrected p-value < 0.05.

### Statistical analysis

RNA-seq data were analyzed and gene expressions were normalized by the method of trimmed mean of M-values (TMM) and glm using the edgeR package, respectively. This yielded a total of 13,575 genes. KEGG pathway enrichment analysis cut-off criteria of FDR<0.05 with a |FC|>2 was apply. RT-PCR data were analyzed with student's unpaired t-test (2-tailed) or one-way ANOVA with post-Bonferroni’s test for multiple comparisons. P≤ 0.05 was considered significant. Data are presented as Mean ± SEM from cells with n≥ 6 (as indicated in figure legends). All data analysis was done in GraphPad Prism or Microsoft Excel.
